# Disabilities Among Newly Reported Leprosy Cases in Visakhapatnam District, India: A Cross-Sectional Study of Prevalence and Associated Factors

**DOI:** 10.7759/cureus.85207

**Published:** 2025-06-01

**Authors:** Siva Priya Jalakam Venkata, Sivakumar Lotheti, Devi Madhavi Bhimarasetty

**Affiliations:** 1 Community Medicine, Panimalar Medical College Hospital and Research Institute, Chennai, IND; 2 Community Medicine, Government Medical College, Vizianagaram, Vizianagaram, IND

**Keywords:** grade 2 disability, leprosy, multibacillary, national leprosy eradication program, stigma, visakhapatnam

## Abstract

Introduction: This study evaluated disabilities among newly diagnosed leprosy cases in Visakhapatnam District, India, following the 2019 Leprosy Case Detection Campaign (LCDC). We aimed to quantify disability prevalence, classified by the World Health Organization (WHO) grades (0, 1, and 2), and identify associated sociodemographic and clinical factors.

Materials and methods: A cross-sectional analytical study included 68 out of 299 newly reported cases of leprosy selected via simple random sampling. Data were collected through clinical examinations, nerve assessments, and patient interviews after informed consent. Disability was graded per WHO criteria.

Results: Participants (mean age: 36.7 ± 18.0 years) included 62% males and 81% aged 15-60 years; 39% were middle-class. Multibacillary leprosy predominated (63%), with 10% of child cases (<15 years), indicating active transmission. Disabilities were observed in 14% of the leprosy cases: 6% grade 1 and 9% grade 2 (exceeding India's 7.7% grade 2 average). Hands were most commonly affected (9%), with ulnar nerve involvement in 62% of the patients. Median healthcare-seeking delay was eight months, correlating with the proportion of grade 2 disabilities (G2D) observed. Stigma was a noted barrier, with patients reporting concealment of symptoms.

Conclusions: Despite LCDC, high G2D rates persist, driven by delayed diagnosis and stigma. Recommendations include enhanced information education and communication, active case detection, and post-multidrug therapy follow-up. These findings support India's National Leprosy Eradication Program and global efforts to reduce leprosy-related disabilities.

## Introduction

Leprosy, caused by *Mycobacterium leprae*, remains a global health concern despite curative multidrug therapy (MDT), with 202,185 new cases reported worldwide in 2019, 58% from India [1]. Peripheral nerve damage leads to disabilities, classified by the World Health Organization (WHO) as grade 0 (no disability), grade 1 (loss of sensation), and grade 2 (visible deformities like claw hand or ulcers) [2]. In India, the National Leprosy Eradication Program (NLEP), launched in 1983, reduced prevalence to 0.61 by 2019, but the grade 2 disabilities (G2D) rate persists at 2.6 per million population [3]. Delayed diagnosis exacerbates irreversible impairments, perpetuating stigma and social exclusion [4]. Leprosy Case Detection Campaign (LCDC) strategies, including house-to-house surveys and Accredited Social Health Activist-based surveillance, aim to reduce G2D to 1 million, aligning with WHO’s 2016-2020 Global Leprosy Strategy [5]. However, post-LCDC data on disability prevalence and associated factors in high-endemic areas like Visakhapatnam District are limited. Studies elsewhere report varying G2D rates, 31.6% in Kerala [6], suggesting regional disparities. Child cases (15 years) indicate active community transmission, while multibacillary leprosy and delayed care increase disability risk [7]. Stigma, rooted in misconceptions like leprosy being a “curse,” further delays diagnosis [8]. Very little literature is available regarding the burden of leprosy and its disabilities after the implementation of the LCDC. Hence the present study was taken up with an aim to assess the G2D rate in Visakhapatnam District after implementation of LCDC with the following study objectives: (1) to determine the proportion of disabilities among newly reported leprosy cases in Visakhapatnam District, (2) to classify disabilities by WHO grades, and (3) to identify sociodemographic and illness-related factors linked to G2D.

## Materials and methods

**Figure 1:**
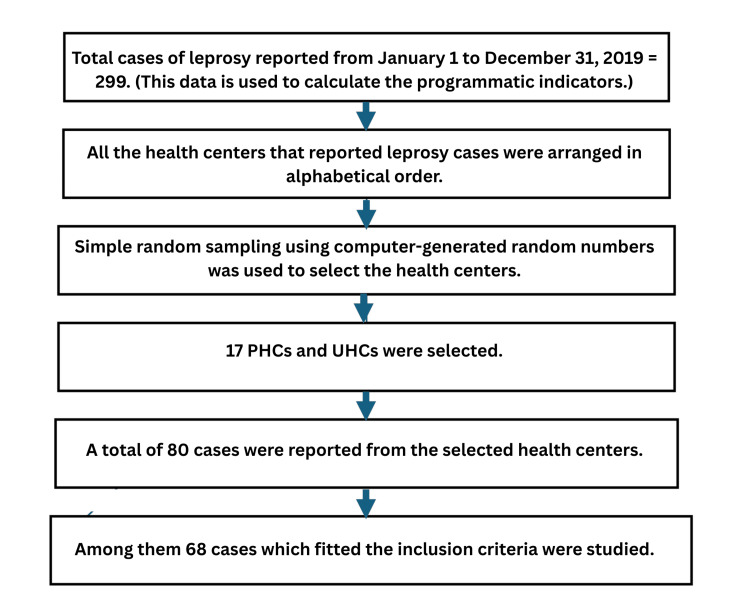
Flowchart showing the sampling technique used in the present study PHCs: primary health centers, UHCs: urban health centers

This observational cross-sectional study was conducted for one year in Visakhapatnam District, a coastal district with a population of ~4.3 million. It has urban, rural, and tribal areas targeted by the LCDC since 2018. The sample size was calculated based on the NLEP Annual Report 2016-2017, in which the prevalence of disabilities among newly reported cases was 12% [9]. By applying the formula n = 4 PQ / L², the sample size is derived as 64 (P = 12% (29), Q = 88%, L = allowable error of 8%). The final sample size is 68 in the present study. For assessing the programmatic indicators, the details of all the 299 newly reported cases registered with the District Leprosy Office for 2019, from January 1 to December 31, were analyzed (Figure 1).

All participants were contacted via mobile phones prior to visiting the primary health center (PHC), and those available on the scheduled day were included in the study. The exclusion criteria comprised registered individuals who remained unavailable after two visits or phone calls and those transferred in or out from other districts. Data were collected using several methods. Clinical examinations were conducted by trained medical officers at the respective PHCs following WHO protocols [2]. Sensory testing was performed using a ballpoint pen to detect loss of sensation on the palms and soles. Voluntary muscle testing assessed muscle strength, graded as strong, weak, or paralyzed, in the facial, ulnar, median, radial, and peroneal nerves. Nerve palpation was used to evaluate thickening or tenderness in the ulnar, median, radial cutaneous, lateral popliteal, and posterior tibial nerves. Case proformas documented sociodemographic details such as age, gender, caste, and socioeconomic status (as per the Modified B.G. Prasad Classification updated for 2018), along with clinical characteristics including leprosy type (paucibacillary (PB) or multibacillary (MB)), presence of lepra reactions, nerve involvement, and disability grades. Semi-structured interviews were conducted with patients to explore delays in seeking healthcare, stigma, and beliefs, with responses recorded verbatim for qualitative analysis, particularly in cases with G2D. A review of records from the District Leprosy Office registers also provided data on diagnosis dates, initiation of MDT, and other programmatic indicators.

For disability assessment, the WHO grading was applied. Grade 0 indicates normal sensation and muscle power; grade 1 denotes loss of sensation without visible deformity; and grade 2 includes visible deformities (e.g., claw hand, ulcers, foot drop) or severe vision loss (6/60). G2D cases underwent detailed investigation using NLEP’s G2D case format, which was adapted from CLTRI Chengalpattu and modified to suit local needs, noting the type of deformity, its progression, and access to healthcare. Data were double-entered into Excel (Microsoft Corp., Redmond, WA, USA) and cross-verified.

Statistical analysis for descriptive statistics included proportions for categorical variables (e.g., disability grades, gender), means ± standard deviation for continuous variables (e.g., age), and medians for non-normally distributed data (e.g., delay duration). No multivariate analysis was performed due to sample size constraints.

Quality control was ensured by standardizing examinations according to NLEP training manuals [2]. Approval was obtained from the Institutional Ethics Committee of King George Hospital (approval number: 147/IEC KGH/SEP/2019), and the district leprosy officer granted permission. Informed written and oral consent was obtained from all participants aged over 18 years. For participants below 18 years of age, informed oral and written consent was obtained from their parents or guardians. Confidentiality of all study participants was maintained.

## Results

Of the 68 participants included in the study, 55 (81%) were aged 15-60 years, with a mean age of 36.72 ± 17.99 years (range 5-76). Seven (10%) cases were children (<15 years), indicating active transmission. Males comprised 42 (62%), and 27 (39%) belonged to the middle socioeconomic class per Modified B.G. Prasad Classification (Table 1). Three (4%) cases reported a family history of leprosy, suggesting household transmission risk.

**Table 1:** Sociodemographic characteristics of the study population (N=68) The Modified B.G. Prasad's Classification for the year 2018 categorizes per capita monthly income as follows: upper class (I) with more than Rs. 6,528, upper middle class (II) from Rs. 3,264 to Rs. 6,527, middle class (III) from Rs. 1,959 to Rs. 3,263, lower middle class (IV) from Rs. 979 to Rs. 1,958, and lower class (V) with less than Rs. 979.

Categories		N (%)
Age in years	≤14	7 (10)
	15-30	22 (32)
	31-45	22 (32)
	45-60	11 (17)
	>60	6 (9)
Gender	Female	26 (38)
	Male	42 (62)
Socioeconomic status (Modified B.G. Prasad Classification updated for 2018)	Upper class I	3 (4.3)
	Upper middle II	21 (30.4)
	Middle III	27 (39.1)
	Lower middle IV	17 (24.6)
	Lower V	1 (1.4)

Clinically, 43 (63%) had MB leprosy, while 25 (37%) had PB. The median delay from symptom onset to healthcare-seeking was eight months (range 1-24), driven by stigma or unawareness. Ulnar nerve involvement was predominant, affecting 42 (62%), followed by the lateral popliteal nerve, affecting 12 (18%) cases. Lepra reactions occurred in a total of seven cases; among them, four (6%) had type 1 and three (4%) had type 2, often linked to nerve damage (Table 2).

**Table 2:** Clinical characteristics of the study population (N=68) MB: multibacillary, PB: paucibacillary

Clinical features		N (%)
Classification	MB leprosy	43 (63)
	PB leprosy	25 (37)
Reaction	No	61 (90)
	Type 1	4 (6)
	Type 2	3 (4)
No of nerves	Nil	23 (34)
	1	10 (15)
	2	19 (28)
	3	9 (13)
	4	6 (9)
	5	1 (1)

Disability assessment revealed 59 (86%) with grade 0, four (6%) with grade 1, and five (9%) with G2D. The G2D rate (5, 9%) exceeded India’s 2018-19 average (7.7%) [3]. Hands were most affected (6, 9%), followed by feet (3, 4.5%), with no eye involvement. Loss of sensation was the most common disability (5, 7.5%), with one G2D case showing multiple deformities (ulcer, digit absorption, claw hand). Four G2D cases had delayed healthcare-seeking (six months), but MDT initiation was timely, with four cases examined during treatment. One case underwent reconstructive surgery for the claw hand, and two were scheduled for surgery (Table 3).

**Table 3:** Distribution of the study population according to WHO disability grade, site, and nature of disability WHO: World Health Organization

WHO disability grading	No. (%)
Grade 0	59 (86)
Grade 1	4 (6)
Grade 2	5 (9)
Site of disability	
Hands	6 (9)
Feet	3 (4)
Eyes	0
Nature of disability	
Hands	6 (9)
Feet	3 (4.5)
No disability	59 (86.5)

The proportion of G2D among new cases was 2.67%, and the G2D rate per million was 1.72, whereas the national average for the financial year 2018-2019 was 3% and 2.6%, respectively; 48.9% of the newly reported cases were diagnosed to have MB type to 52.3% for the financial year 2018-2019 in India, and 9.4% of the new cases detected are child cases. The detected proportion was more than the national average for 2018-2019, which is 7.7% [3]. Upon analyzing the patients’ narratives, it was understood that stigma is the reason for the delay in seeking healthcare. A 45-year-old male with G2D (claw hand) said, “I hid my patches, fearing rejection by my village. I thought it was just a skin problem.” A 30-year-old female with grade 1 disability noted, “People said leprosy is a curse. I waited a year, scared to tell anyone.” A 50-year-old male with G2D (ulcer) shared, “I didn’t know treatment was free. I spent months with a faith healer first.” These quotes reveal misconceptions (“curse,” “skin problem”) and fear of social ostracism, delaying care, and worsening outcomes.

## Discussion

The G2D rate noted in the current study is 9%, which exceeds India’s 7.7% average for 2018-2019 [3], with 63% having MB leprosy. Similarly, Srinivas et al.’s case-control study across five Indian states found that MB leprosy increased disability risk (OR 9.1) [7], reinforcing our findings. However, our G2D rate is lower than Reyila et al.’s 31.6% in Kerala [6], where borderline tuberculoid cases predominated, unlike our MB-heavy sample. These disparities suggest regional variations in case detection and clinical presentation. The seven (10%) child cases signal active transmission, consistent with NLEP’s concern for ongoing community spread [3]. Our study had no children with disabilities, likely due to early PB leprosy diagnosis. However, child cases underscore the need for contact tracing and post-exposure prophylaxis, which NLEP has implemented since 2018 [10]. The eight-month median healthcare-seeking delay, linked to G2D in five cases, mirrors Srinivas et al.’s finding, highlighting that a three-month delay increased disability risk (OR 1.6) among the leprosy cases [7]. Qualitative data supports the quantitative burden of disability. One patient’s statement, “I concealed my patches to avoid rejection,” highlights the role of stigma, mirroring Govindharaj et al.’s finding that 78% of leprosy patients feared disclosure [11]. Another comment, “I assumed it was just a skin issue,” reveals limited awareness, aligning with Saha et al.’s KAP study, where 79% lacked leprosy knowledge before diagnosis [12]. Hand predominance in G2D, seen in six (9%) cases, corresponds with Rathod et al.’s 44.48% hand deformity rate [13], affecting function, as one patient stated, “I struggle to grip tools now.” The absence of eye involvement differs from Reyila et al.’s facial nerve palsy cases [6], possibly due to our limited G2D sample or examination scope. No treatment delays occurred, with all G2D cases receiving timely MDT, reflecting LCDC’s effective case linkage, in contrast to Muthuvel et al.’s noted health system delays [14]. Yet, stigma persisted; one patient’s belief that “leprosy is a curse” led to a faith healer, delaying care by a year. Compared to global standards, our G2D rate matches WHO’s 2019 report of 10% disability in new Southeast Asian cases [1]. However, meeting WHO’s 1 million G2D target demands tackling delays and stigma, as one patient’s remark, “I didn’t know treatment was free,” indicates gaps in information, education, and communication. NLEP’s LCDC, initiated in 2017 [10], should enhance community outreach, using mascots like SAPNA to counter myths, as five G2D cases cited fear or ignorance. The study’s quality would have improved with examinations at MDT’s start and end to better track disability progression. Additionally, omitting comorbidities like diabetes, pregnancy, or other medical conditions that could influence MDT use and disability development was a limitation.

## Conclusions

The study on the proportion of disabilities among newly reported leprosy showed a higher proportion of G2D, with five (9%) cases at the time of examination, which will impair the functionality in carrying out routine activities. Hands are the most common site of disability, seen in six (9%) individuals. Seven (10%) of them are child cases, indicating active community transmission. The most involved nerve was the ulnar, affecting 42 (62%) of the study population. MB leprosy was found to be associated with the development of disability. Satisfactory performance was noted in terms of case detection. At the individual and community level, misconceptions and a lack of awareness regarding leprosy were noted. LCDC facilitated timely MDT, but persistent misconceptions demand robust IEC.

## Appendices

**Table 4:** Case proforma PHC: primary health center, SC: scheduled caste, ST: scheduled tribe, PB: paucibacillary, MB: multibacillary, RFT: release from treatment, WHO: World Health Organization

Case proforma – clinical examination			
Name:		PHC:	
Address:		Age:	
Sex:	☐ Male		☐ Female
Category:	☐ SC	☐ ST	☐ Others
Classification:	☐ PB		☐ MB
Visible deformity:	☐ Yes		☐ No
Date of first dose:			
History of lepra reactions/nerve examination			
RFT:			
Others (specify):			
Neurological and disability assessment			
Side	Voluntary muscle testing and disability grading	Side	
Right		Left	
Date:			
Vision (0, 2):			
Light closure lid gap (in mm):			
Blink (present/absent):			
Little finger out:			
Thumb up:			
Wrist extension:			
Foot up:			
Disability grade – hands:			
Disability grade – feet:			
Disability grade – eyes:			
Max. (WHO) disability grade:			
Sensory testing			
Date/assessor			
	Palm	Sole	Comments
Right			
Left			

**Table 5:** NLEP G2D case investigation format CHC: community health center, PHC: primary health center, H/o: history of, MDT: multidrug therapy, MB: multibacillary, PB: paucibacillary, NLEP: National Leprosy Eradication Program, G2D: grade 2 disability

State:
District:
Block:
CHC:
PHC:
Gram panchayat:
Municipality:
Street/ward:

Name:
Gender:
Address:
Education:
Occupation:
Name of the family member and relation to the patient:
Date of onset of skin nerve changes:
Description of deformity that happened after the damage: minor cracks/wound/ulcer/scar/contractures/clawing/changes in hand and foot, or their falling off/others specify _____________
H/o progression of deformity:
Features of leprosy in eyes: yes/no
If yes, which of the following are present: diminition of vision/unable to do finger counting/lagophtalmous/reduced or absent blinking of eyes/visible gap after closing both eyes/others specify __
Date of confirmation of diagnosis of leprosy:
Where was leprosy diagnosed: government/private:
Was a skin smear taken:
Date of initiation of MDT: (When was the first dose given)
Date of provision of other doses of MDT:
H/o missed dose:
Reason for missed dose:
Did you undergo any examination by a doctor during the course of MDT: yes/no

Practices related to health:
a. What is the faith of quacks regarding treatment?
b. Reason for not revealing the disease to the health worker who visited your village?
c. Reason for not approaching the health facility?
H/o migration in past 3 years: yes/no
Was treatment stopped during migration: yes/no

Date of diagnosis	Place of diagnosis	Classification MB/PB	Grade 1/2
